# Recovering from the Unprecedented Backsliding in Immunization Coverage: Learnings from Country Programming in Five Countries through the Past Two Years of COVID-19 Pandemic Disruptions

**DOI:** 10.3390/vaccines11020375

**Published:** 2023-02-07

**Authors:** Anithasree Athiyaman, Tosin Ajayi, Faith Mutuku, Fredrick Luwaga, Sarah Bryer, Omotayo Giwa, Shadrack Mngemane, Nnang Nadege Edwige, Leslie Berman

**Affiliations:** Clinton Health Access Initiative (CHAI), Global Vaccines Delivery Team and Country Offices, Boston, MA 02127, USA

**Keywords:** routine immunization, COVID-19 pandemic, coverage backsliding, recovery, Cambodia, Cameroon, Kenya, Nigeria, Uganda

## Abstract

Between 2020 and 2021, the COVID-19 pandemic severely strained health systems across countries, leaving millions without access to essential healthcare services. Immunization programs experienced a ‘double burden’ of challenges: initial pandemic-related lockdowns disrupted access to routine immunization services, while subsequent COVID-19 vaccination efforts shifted often limited resources away from routine services. The latest World Health Organization (WHO) and United Nations Children’s Fund (UNICEF) estimates suggest that 25 million children did not receive routine vaccinations in 2021, six million more than in 2019 and the highest number witnessed in nearly two decades. Recovering from this sobering setback requires a united push on several fronts. Intensifying the catch-up of routine immunization services is critical to reach children left behind during the pandemic and bridge large immunity gaps in countries. At the same time, we must strengthen the resilience of immunization systems to withstand future pandemics if we hope to achieve the goals of Immunization Agenda 2030 to ensure vaccinations are available for everyone, everywhere by 2030. In this article, leveraging the key actions for sustainable global immunization progress as a framework, we spotlight examples of strategies used by five countries—Cambodia, Cameroon, Kenya, Nigeria, and Uganda—who have exhibited exemplar performance in strengthening routine immunization programs and restored lost coverage levels in the last two years of the COVID-19 pandemic. The contents of this article will be helpful for countries seeking to maintain, restore, and strengthen their immunization services and catch up missed children in the context of pandemic recovery and to direct their focus toward building back a better resilience of their immunization systems to respond more rapidly and effectively, despite new and emerging challenges.

## 1. Introduction

Between 2020 and 2021, the COVID-19 pandemic severely strained health systems across countries, leaving millions without access to essential healthcare services. Routine Immunization programs experienced a ‘double burden’ of challenges due to the COVID-19 pandemic and associated disruptions and to the subsequent COVID-19 vaccination efforts shifting often limited resources away from routine services. The latest World Health Organization (WHO) and United Nations Children’s Fund (UNICEF) estimates suggest that 25 million children missed routine vaccinations in 2021, six million more than in 2019 and the highest number witnessed in nearly two decades.

Recovering from this sobering setback requires a united push on several fronts. Intensifying the catch-up of routine immunization services is critical to reach children left behind during the pandemic and bridge large immunity gaps in countries. At the same time, we must strengthen the resilience of immunization systems to withstand future pandemics if we hope to achieve the goals of Immunization Agenda 2030 to ensure vaccinations are available for everyone, everywhere by 2030.

## 2. Materials and Methods

This article spotlights examples of strategies used by five countries—Cambodia, Cameroon, Kenya, Nigeria, and Uganda—that have exhibited exemplar performance in strengthening routine immunization programs and that have restored lost coverage levels back to pre-pandemic levels to identify country-level learnings and inform and support other countries to adopt similar practices. No personal or patient-level health information was gathered for this work, and this work is not considered human subjects research. Coverage data was obtained from WHO-UNICEF and reported at the country-level. 

## 3. Discussions

### 3.1. The Challenge: Historic Backsliding in Routine Immunization Coverage

Across the world, the COVID-19 pandemic has disrupted essential health services, adversely impacting significant gains in health outcomes achieved in recent decades. This is particularly so for routine immunizations, with recently published WHO-UNICEF estimates showing historic reductions in immunization coverage in 2021, with 25 million children missing out on life-saving vaccines, the highest number since 2006 [1]. The number of ‘zero dose children’ (those who did not receive any dose of Diphtheria, Tetanus and Pertussis (DTP) containing vaccines) [2] increased sharply from 13 to 18 million during the pandemic period, a shocking 37% increase since 2019. While immunization coverage dropped in all WHO regions, some regions were more affected than others, with coverage dips ranging between 1% in Europe to as high as 9% in South-East Asia.

As a result of this monumental drop in coverage, a disproportionate number of children in low- and middle-income countries are left most vulnerable to vaccine-preventable diseases. With rising immunity gaps, the risk of large outbreaks is imminent, with cases of measles already reported in Africa and Eastern Mediterranean, and wild polio virus 1 (WPV1) detected outside the endemic countries in Asia [3]. This threatens the lives of unprotected children and could be severely disruptive to already over-stretched health services. Furthermore, some specific newly introduced antigens like Human Papillomavirus (HPV) vaccines are also seen to be more adversely impacted and less resilient to these shocks. Over the last two years, HPV vaccine coverage dropped by 15% [1]. Since 2019, 3.5 million eligible girls have not yet received their first dose of HPV vaccine, the highest decline since 2010. The trend is compounded by the fact that 59% of cervical cancer cases occur in countries that have not yet introduced HPV vaccination into their national programs, leaving millions of adolescent girls unprotected against cervical cancer.

Routine immunization programs were impacted by a ‘double burden’ of managing the disruptive effect of the COVID-19 pandemic as well as the subsequent historic COVID-19 vaccination efforts to improve population immunity against the virus. Nearly all countries reported some form of disruption to routine immunization (RI) services in 2020 and early 2021, with primary and community care among the most affected service delivery settings [4]. In many countries, several planned outreaches and even Supplementary Immunization Activity (SIA) were either suspended or postponed. In early 2021, more than one third of countries (37%) participating in surveys monitoring pandemic impact on health systems still reported disruptions to their routine immunization services in comparison to early 2020 [4].

Despite these sobering trends, some countries improved vaccination coverage during the pandemic—39 countries recovered or almost recovered to pre-pandemic levels in 2021. But over two years, only 24 countries achieved higher coverage in 2021 than in 2019 [5]. This notable progress is attributable to the intensification and mitigation efforts of country programs to maintain and/or resume vaccine delivery to catch up on missed populations. In some cases, these countries have managed to also attain high COVID-19 vaccination coverage—the fastest and one of the most complex global vaccine campaigns in history.

### 3.2. The Opportunity: Restoring Coverage and Strengthening Program Resilience

The vulnerability of global immunization systems demonstrates the urgent need to maintain, restore, and strengthen routine immunization systems in order to address the widening immunity gap in the context of the pandemic and build resilience for future shocks. Despite the complexities of managing immunization programs during a continually evolving pandemic, several countries have shown that improving immunization coverage is possible. Documenting and spotlighting learnings from these countries will help other countries to adopt similar practices to increase vaccination equity.

**Figure 1:**
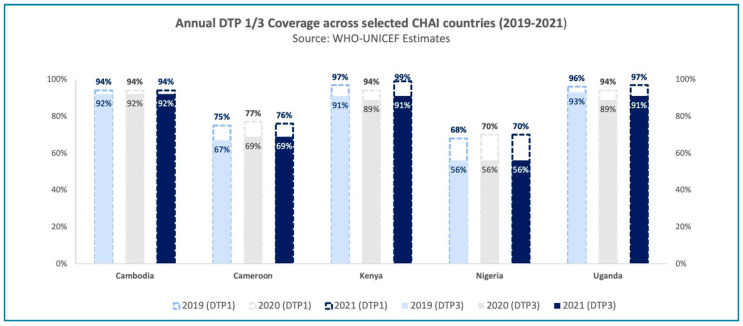
Graph representing DTP1/3 coverage levels in selected program countries—Cambodia, Cameroon, Kenya, Nigeria, and Uganda—between 2019 and 2021. It highlights that across these five countries, national coverage of DTP3 remained the same or increased between 2019 and 2021, showcasing the immunization systems’ resilience to withstand shocks due to the COVID-19 pandemic. Source: 2021 WHO-UNICEF Estimates.

This article presents examples from five selected countries—Cambodia, Cameroon, Kenya, Nigeria, and Uganda—that have either maintained or increased their Diphtheria, Tetanus, and Pertussis–containing vaccine (DTP3) coverage rates in 2021 compared to 2019/20 (see Figure 1). Through strong partnership with the Ministries of Health in these countries, CHAI and immunization partners have witnessed and supported their journey to recovery. Using the key actions for sustaining global immunization progress [6], which highlight the urgent steps necessary for sustaining immunization activities globally as a conceptual framework, we outline several exemplary strategies adopted by these countries over the past two years that contributed to favorable immunization outcomes—also see [File S1].

#### 3.2.1. Conducting Frequent and Intensified Catch-up Activities

Closing immunity gaps and reaching missed communities require intensified efforts that are well planned and informed by evidence. In Cambodia, Kenya, Nigeria, Cameroon, and Uganda, multiple rounds of catch-up activities were conducted to service high-risk communities throughout the pandemic to address coverage gaps and inequities. These included a mix of intensified and targeted outreaches and campaign style periodic intensification of routine immunization (PIRIs) activities e.g., integrated child health days (ICHDs) and local immunization days (LIDs). In Cameroon, districts in conflict-affected regions were serviced through three rounds of periodic intensifications of routine immunizations (PIRI), which resulted in 28% improvement in DTP3 coverage in the South West Region in 2020/2021. To support with planning these outreach efforts, Clinton Health Access Initiative (CHAI) Cameroon helped track on a quarterly basis district level service delivery and immunization coverage indicators. We used an Excel/power BI-based dashboard, which enabled the rapid identification and prioritization of districts that required intensified catch-up activities. Similarly, in Cambodia, CHAI supported with data review and field assessments to assess and update the Expanded Programme for Immunization (EPI) list of high-risk communities with recent pockets of missed children, while in Kenya, CHAI supported the roll-out of tracking tools to monitor PIRI microplanning progress and review the accuracy and completion of immunization micro plans. In Nigeria, CHAI supported program planning for a catch-up campaign and identifying zero-dose children in 145 districts across the country, including redesigning microplanning and other data tools to accommodate infection prevention and control (IPC) needs in the context of the pandemic.

#### 3.2.2. Strengthening Health Information Systems to Routinely Capture Immunization Coverage and Ongoing Disease Surveillance

Despite challenges brought on by the pandemic, these countries continued to strengthen health information system capacities to capture and use routine immunization data for planning and implementation. CHAI provided technical support to EPI across all levels in Cambodia, Uganda, and Kenya to strengthen data management and review capacities to promote immunization data use for planning and decision making. By promoting a systemic approach that leverages existing data, underserved communities can be identified and necessary resources allocated for rapid course correction. In Cambodia, CHAI supported the development of a new visualization dashboard that provided easy access and review of coverage gaps at all levels (national, provincial, district, and service delivery point) to enable prompt follow-up and action. The dashboard was made available in English and the local language, Khmer, for ease of access and user-friendliness. In Kenya, CHAI was instrumental in supporting the Health Management Information System (HMIS) team in separating PIRI and RI indicators within the DHIS2 platform to enable clear performance monitoring for supplementary immunization activities. In Cameroon, CHAI supported the identification and characterization of zero-dose communities using triangulation of demographic, geographic, and immunization data. Through this effort, health areas with the highest risk or probability of zero-dose children were prioritized for targeted action. CHAI also supported mentoring activities to improve data completeness, timeliness, and quality into DHIS2, resulting in an increase of 18% in timeliness, 5% in data quality, and 5% in completeness in the Adamawa region in Cameroon. In Nigeria, to inform decision making, CHAI was instrumental in the roll out of the PowerBI tool for the visualization of real-time campaign immunization coverage and reach. In Uganda, CHAI strengthened identification of underserved areas within health facility catchment areas through monthly reviews of health facility immunization registration data, resulting in in ~50% increase in the number of children from underserved villages vaccinated against DTP3 and MR1 [7].

#### 3.2.3. Finding Synergies with the COVID-19 Vaccine Roll-out

Across the spectrum of activities undertaken to plan, implement, and monitor the COVID-19 vaccine roll-out, many of the countries who demonstrated resilience in the last two years capitalized on these activities for the mutual benefit of routine immunizations.

- To improve integrated delivery of services and promote a life course approach to vaccination, the Cambodian Ministry of Health, with support from CHAI and other partners, developed policies to integrate routine immunization into the COVID-19 outreach strategy in 2021, with a focus on prioritizing hard-to-reach communities. CHAI’s support included the following:
  ○ The design and implementation of an integrated NCD screening and COVID-19 vaccination pilot in two provinces, which was initially implemented at mass vaccination sites and later shifted to health center fixed sites after the acute emergency phase (as booster dose delivery picked up pace). In the first five months during implementation at mass vaccination sites, the pilot successfully resulted in the referral or linkage to care for approximately ~1600 adults with previously undiagnosed diabetes or hypertension. By the end of the second phase of the pilot (February 2022), which has now been transitioned to government implementation, a total of ~2700 adults had been referred for appropriate follow-up care.
  ○ To optimize healthcare worker capacity and national health budgets to provide hard-to-reach communities with immunization services, CHAI supported with analyzing COVID-19 and routine immunization coverage data at the provincial, district, and village levels to inform the implementation of such integrated outreach in previously underserved communities.
- The Ugandan Ministry of Health, with support from partners, developed and disseminated operational guidelines to support the symbiotic delivery of health services alongside COVID-19 vaccination, care, and treatment. This included models that encourage task shifting among healthcare workers and hybrid offsite and onsite approaches to supportive supervision to better balance workload and delivery of health services at the district level.
- Data reporting helped countries recognize and react to the cannibalistic effect COVID-19 vaccination was having on healthcare workers’ ability to deliver other primary healthcare (PHC) services, including routine immunization. The government of Nigeria decided to separate the service delivery and data collection functions of COVID-19 vaccination to support front line healthcare workers to prioritize the dual duty of COVID vaccinations and other PHC services. CHAI provided guidance in developing strategies and adopting technical guidelines for the integration of COVID-19 vaccination with other PHC services, including immunization, Vitamin-A, and ante-natal care (ANC) services. Three main strategies were promoted, which combined to boost coverage by 70% by October 2022. The strategies included the following:
  ○ TEACH, which combines traditional microplanning methods with appropriate technology.
  ○ A family-centered integrated PHC approach that translates into the national strategy of improving access to basic health services.
  ○ SCALES 3.0 (Supervision, Communication, Accountability, Logistics, Immunization data and Service delivery), a strategy that incorporates integration of services, performance-based incentives, data use for action, and decentralized demand generation.
- In Cameroon, Kenya, and Nigeria, COVID-19 vaccination training was also used to refresh frontline healthcare worker knowledge on routine immunization and promote broader immunization best practices.
- In Kenya, through support from CHAI, several county-level EPIs leveraged COVID-19 vaccine outreach services, targeting teachers in schools to co-deliver HPV vaccinations to eligible adolescent girls enrolled in the schools.

#### 3.2.4. Mobilizing Resources for Sustaining Immunization Services

Early intervention by governments to provide clear directives to health facilities was instrumental in minimizing service delivery disruptions. For example, the mid-2020 nationwide directive in Nigeria to continue routine outreach sessions resulted in an 11.6% increase [8] in service provision since the early disruptions. In Uganda, by engaging non-traditional health stakeholders, particularly at the district level, the immunization program was able to mobilize additional financing and resources, including human resources to support data management and operational aspects of delivering vaccinations, which tremendously eased pressure on system capacities and helped sustain routine immunization services.

#### 3.2.5. Restructuring Health Systems to Build Resilience

Learning from the largest disruption to immunization service delivery in three decades and the largest vaccination rollout in history, a few countries are prioritizing investments in systems that enable multisectoral collaboration with strong community participation for agile decision making. For example, the Cambodian government is in the process of developing a PHC booster strategy to ensure all individuals can access a quality package of care in the public sector—from prevention to early diagnosis and management across the life course, encompassing maternal and child health, communicable diseases, selected non-communicable diseases, mental health, and other ageing-related illnesses. This strategy aims to emphasize stronger community engagement and new models of service delivery that could strengthen the resilience of health systems. CHAI is supporting by updating the community participation policy, which will redefine the governance, roles, and responsibilities for community heath workers within existing community structures. In Uganda, the government is adapting routine immunization for the use of Smart Paper Technology, which enables individual-level tracking of COVID-19 vaccine recipients and includes a reminder function for subsequent doses. CHAI is supporting the country to develop systems and processes that reduce missed opportunities for vaccination at every encounter with the health system, which has had a demonstrable impact on all antigens of note, including sustained increase in the number of vaccinated (11% in DTP3, 4% in MCV1, 72% in HPV2) in the supported districts in 2021 [7]. These health system capacities and processes will facilitate improved preparedness and operations to rapidly respond to emergent system shocks while maintaining the effectiveness of routine programs.

## 4. Conclusions

The importance of integrating routine immunization into primary healthcare systems has never been clearer. As countries continue to recover and adopt lessons from the last few years, to not only mitigate the effects of backsliding but to reach those who were previously unreached, integrated and holistic PHC systems offer the best way forward for supporting resilient and sustainable routine immunization programs. Investing in broader health system strengthening and improving linkages with communities will help advance immunization goals. With the future of the COVID-19 pandemic remaining uncertain, it is vital to focus on building back better the resilience of our immunization systems to respond more rapidly and effectively to challenges, and ensure all children continue to have access to lifesaving vaccinations despite new and emerging concerns. Learnings from the exemplary countries presented in this article provide insights into the various possibilities that can be unlocked with the right commitment and support.

## Data Availability

No new data were created or analyzed in this study. Data sharing is not applicable to this article.

## Supplementary Materials

The following supporting information can be downloaded at: https://www.mdpi.com/article/10.3390/vaccines11020375/s1, File S1: Enablers for RI Recovery in CHAI focal countries.
