# Supplementary material for: Recovering from the Unprecedented Backsliding in Immunization Coverage: Learnings from Country Programming in Five Countries through the Past Two Years of COVID-19 Pandemic Disruptions
Source: Vaccines (Basel). 2023 Feb 7;11(2):375. doi: 10.3390/vaccines11020375 (PMC9960337; doi:10.3390/vaccines11020375)
Supplement: Supplementary file 1 [file vaccines-11-00375-s001.zip › vaccines-2063383-supplementary.pdf]

CLINTON HEALTH ACCESS INITIATIVE

Key drivers for sustaining immunization progress in 2020-2021 across selected CHAI-supported countries: Cambodia, Cameroon, Kenya, Nigeria and Uganda

Consolidation of preliminary findings

August 2022

Key drivers for sustaining immunization progress in 2020-2021 (1/4)

Examples from selected CHAI-supported countries

Thematic Area | **Key Actions Undertaken**

CHAI supported activities are highlighted in blue

Frequency and approach of catch-up vaccinations used

Cambodia

- Cambodia conducted two to four rounds of catch-up outreach activities in all districts throughout the pandemic (2020-2022).
- CHAI supported NIP to conduct data reviews and field assessments as part of the development of Cambodia's strategic Full Portfolio Plan (FPP) application to secure Gavi funding. These field assessments were critical in updating NIP's list of high-risk communities with recent pockets of missed communities which informed catch up outreach planning.

Cameroon

- Cameroon EPI conducted three rounds of Periodic Intensification of Routine Immunization (PIRI) and disease surveillance per health district in conflict affected regions - South-west and North-west. Through this, in South-west region alone, over 54,000 children were vaccinated, contributing to a 28-percentage point increase in regional coverage.
- To improve coverage and equity, EPI with support from partners conducted microplanning and outreach intensification in 54 low performing health districts.
- With support from CHAI, the Cameroon EPI tracked on a quarterly basis district level service delivery and immunization coverage indicators using an excel/power BI based dashboard that enabled the rapid identification and prioritization for action districts that required intensified catch-up activities

Kenya

- Kenya conducted several rounds of catch-up vaccinations to reach those that had been previously missed and close any coverage gaps. National PIRIs were conducted in all 47 counties and MR Supplemental Immunization Activities (SIA) conducted in 22 high risk counties, with all data captured in DHIS2.
- CHAI supported the roll out of PIRI microplanning progress tracker to support with the review of accuracy and completion rates of micro plans developed.

Nigeria

- With support from CHAI and other partners, NPHCDA carried out key activities and strategies to mitigate the effect of the pandemic on RI, including:1) multiple rounds of catch-up and outreach services to high-risk communities 2) PIRI activities in addition to local immunization days (LIDs) 3) annual engagement with selected low performing LGAs (high priority/zero dose LGAs), 4) Optimized Integrated Routine Immunization Services (OIRIS) visits every two months 5) monthly supportive supervision visits
- Furthermore, CHAI conducted an analysis on sustainability of RI/PHC efforts during the pandemic. These findings were utilized to develop/scale up robust strategies to address the eminent challenges in RI/PHC service delivery.

Uganda

- With support from CHAI and other partners, UNEPI planned and executed three rounds of the biannual integrated child health days (ICHDs) which were conducted in April and October 2021/22
- In addition, CHAI supported the development of immunization catch up plans in focal districts to facilitate coverage recovery; these catchup plans were then adapted and cascaded by UNEPI to other districts.
- CHAI also facilitated the planning and optimization of outreach initiatives by identifying underserved areas within health facility catchment using an innovative approach based on immunization register.
- UNEPI also conducted door-to-door polio vaccination campaign in early 2022 to avoid re-emergency of polio outbreaks.

Key drivers for sustaining immunization progress in 2020-2021 (2/4)

Examples from selected CHAI-supported countries

Thematic Area		Key Actions Undertaken
Strengthening health information systems to routinely capture immunization coverage and ongoing disease surveillance	Cambodia	• In addition to the technical support CHAI provides on promoting immunization data use for planning and decision making by **strengthening data management capabilities** at each level of the health system, In 2021, CHAI supported the development of an **immunization dashboard** using HMIS data to visualize RI program data at all levels (national, provincial, district, and health facility) and enable easy analysis and interpretation (in English and Khmer). The dashboard is used to identify areas where follow up action and support is required.
	Cameroon	• In Cameroon, **CHAI supported mentoring activities** to improve data completeness, timeliness and quality into DHIS2. Central level mentors to provide were upskilled to provide dedicated support to regions, contributing towards improved completeness and timely data reporting in DHIS2. It also led to improvements in data quality as close follow-up was provided to regions with data concerns.
	Kenya	• CHAI provided technical support, working closely with NVIP and HMIS to **integrate data review indicators on KHIS** to ease the process of data reviews, enabling national and county level stakeholders have clear and consistent data visibility for decision making.<br>• During the implementation of PIRIs in Kenya, CHAI supported the GoK HMIS team in **separating PIRI and RI indicators within DHIS2** to enable clear performance monitoring for supplementary immunization activities<br>• Through the continued use of Chanjo eLMIS – **an eLMIS system designed and developed by CHAI**, GoK was able to collect data on stock availability and location for RI and COVID-19 helped maintain healthy and accurate stock pipeline.
	Nigeria	• CHAI supported in conducting an **integrated post-introduction evaluation (IPIE) in 6 selected states** to assess the effect of the previously introduced vaccines (MenA, MCV2, and IPV2) on specific areas of the immunization program. Recommendations were provided for the challenges and gaps. Documented Lessons will improve future NVIs.<br>• CHAI also supported with MICS/ NICS survey to support government and partners to track the pandemic- related impact on immunization coverage.
	Uganda	• Through support from CHAI and other partners e.g WHO, Shifo etc, UNEPI engaged in several interventions to strengthen data collection and use:<br>– Enhanced immunization data reviews and use of data for COVID-19 vaccination at different levels to improve attention towards the challenges in RI. This included routine monitoring of the performance of immunization indicators through DHIS2 data analysis.<br>– Enhanced AEFI follow up structures for COVID-19 have had a ripple effect on RI vaccines.<br>– Enhanced active disease surveillance through supportive supervision<br>• CHAI also supported the use of immunization register to **identify and target underserved communities in a facility's catchment area**, which led to a ~50% increase in the number of children from underserved villages vaccinated against DPT3 and MR1

CHAI supported activities are highlighted in **blue**

Key drivers for sustaining immunization progress in 2020-2021 (3/4)

Examples from selected CHAI-supported countries

Thematic Area | **Key Actions Undertaken**

CHAI supported activities are highlighted in **blue**

Finding synergies with the COVID-19 vaccine rollout

Cambodia

- To improve integrated delivery of services and promote a life course approach to vaccination, The Cambodian government through support from CHAI and other partners e.g. WHO, UNICEF, developed policies to integrate routine immunization into COVID-19 outreach strategy in 2021 with a focus on hard-to-reach communities. Cambodia's outreach strategy is now fully integrated between RI and COVID-19 vaccines.
- CHAI's support included: 1) supporting NIP and provincial EPIs to identify villages/communities with high numbers of unvaccinated or partly vaccinated people for COVID-19 vaccination and mobilize them to vaccination posts as well as **support vaccination teams to conduct integrated outreach to these communities 2) designing and implementing an integrated NCD screening and COVID-19 vaccination pilot in 2 provinces.** More details here

Cameroon

- The Cameroon EPI leveraged the opportunity of COVID-19 vaccination trainings to build healthcare worker capacities on routine immunization best practices.

Kenya

- In Kenya, through support from CHAI, **several county level EPIs leveraged COVID-19 vaccine outreach services** targeting teachers in schools to co-deliver HPV vaccinations to adolescent girls
- Integration of RI and COVID-19 vaccine delivery in hard-to-reach areas was also promoted, with alternatives for quicker and more efficient vaccines delivery being explored e.g., airlifting of COVID-19 vaccines with RI vaccines to Arid and Semi-Arid Lands (ASAL) counties.
- Healthcare worker COVID-19 vaccination training sessions in Kenya were used to refresh frontline healthcare workers knowledge on routine immunization and promote broader immunization best practices.

Nigeria

- CHAI and other partners supported the GoN with the **development of the SCALES 3.0 strategy to accelerate COVID19 vaccination and ramp-up coverage RI and other PHC services**. The main enablers for SCALES 3.0 are intensive campaigns, comprehensive resource and partner mapping, targeted and robust demand generation, strengthened electronic data reporting, a continuous performance management plan for efficiency, and state/context-specific approaches to integrated service delivery.
- The government of Nigeria decided to separate the service delivery and data collection function of COVID-19 vaccination to support front line healthcare workers prioritize the dual duty of COVID vaccinations and other PHC services.

Uganda

- Ugandan MOH with support from partners developed and disseminated operational guidelines to support the symbiotic delivery of health services alongside COVID-19 vaccination, care and treatment. This included models that encourage task shifting among healthcare workers **and hybrid offsite and onsite approaches to supportive supervision** to better balance workload and delivery of health services at district level.

Key drivers for sustaining immunization progress in 2020-2021 (4/4)

Examples from selected CHAI-supported countries

CHAI supported activities are highlighted in blue

Thematic Area	Country	Key Actions Undertaken	Country	Key Actions Undertaken
Mobilizing resources for sustaining immunization services	Cambodia	• Rapid and clear communication from the MOH was issued very early to encourage EPI and HC staff to continue the provision of RI services to minimize disruption to service delivery.<br>• Furthermore, CHAI and other partners provided technical support to NIP in developing synergistic applications for Gavi funding through **Full portfolio planning (FPP) covering 2023-2027, CDS Early Access and CDS3 funding, in an integrated manner that considers the full needs of the RI and COVID vax delivery programs**	Kenya	• CHAI is closely working with NVIP to **mobilize resources for COVID-19 vaccination efforts through CDS funding**, including integrated RI/COVID-19 healthcare worker training and outreaches.
			Nigeria	• The nationwide directive in Nigeria to continue routine outreach sessions resulted in 11.6% increase in service provision since early disruptions.<br>• CHAI supported in **mapping of government/partners resources and areas of support** and developed **state specific accountability framework** to track funds pooled for the vaccination campaign activities in the states.
	Cameroon	• CHAI supported in disseminating findings from the recent procurement mapping and facilitated **advocacy for improved state funding mobilization mechanisms** for timely and adequate vaccines procurement.	Uganda	• UNEPI engaged non-traditional stakeholders particularly at district level, to mobilize financial and human resources to support operational aspects of delivery and data management, in support of COVID-19 vaccinations and sustaining RI services.
Restructuring health systems to build resilience	Cambodia	• Strengthening of community engagement to deliver quality PHC services is a foundational pillar for the new 'PHC Booster' strategy in Cambodia.<br>• CHAI is supporting with **updating the community participation policy (CPP)** which will define the governance and roles of commune health structures.	Kenya	• CHAI conducted **health-facility immunization status survey** to collect data and understand the pandemic-related impact on immunization services and findings were used for planning and decision making on RI service delivery.
			Nigeria	• CHAI supported **NPHCDA trainings of >220,000 HCWs to deliver integrated services**<br>• Integration of SIAs, COVID-19, RI in all fixed posts, VAS in all fixed and temporary fixed Integrate RI in all vaccination posts improved coverage.
	Cameroon	• EPI and partners disseminated guidelines which included the redesign of patients flow in hospitals to ensure continuation of immunization services during a pandemic.	Uganda	• The Ugandan MOH is adapting the Smart Paper Technology used for tracking of individual level COVID-19 vaccinations and sending reminders for subsequent doses for RI.<br>• Furthermore, CHAI is supporting UNEPI to **institutionalize daily vaccinations and improve screening processes in high volume facilities**, resulting in reduced missed opportunities for vaccinations. This resulted in a sustained increase in the number of children vaccinated across all antigens of note (11% DTP3, 4% MCV1, 72% HPV2) in the supported districts in 2021.

For additional information, please contact

Anithasree Athiyaman aathiyaman@clintonhealthaccess.org

Or Tosin Ajayi tajayi@clintonhealthaccess.org

CLINTON HEALTH ACCESS INITIATIVE

www.clintonhealthaccess.org
